# Supplementary material for: Optimizing ECG to detect echocardiographic left ventricular hypertrophy with computer-based ECG data and machine learning
Source: PLoS One. 2021 Nov 30;16(11):e0260661. doi: 10.1371/journal.pone.0260661 (PMC8631676; doi:10.1371/journal.pone.0260661)
Supplement: S1 Table — This table shows a comprehensive explanation of 458 ECG parameters obtained with the Philips DXL-16 algorithm. This high number of ECG parameters allows an extensive analysis of the electricity of the heart. All these measurements are obtained in every lead. (DOCX) [file pone.0260661.s001.docx]

**S1 Table. A list of the ECG parameters analyzed by the Philips DXL-16 algorithm.**

| **Parameter** | **Units or Value** | **Description** | **Type of variable** |
| --- | --- | --- | --- |
| **Morphology lead measurements** | | |  |
| **P AMP** | mV | P wave amplitude | Numeric |
| **P DUR** | msec | P wave duration | Numeric |
| **P AREA** | Ashman units  (40 msec x 0.1 mV) | P wave area for monophasic P waves or the area of the initial portion of a biphasic P wave | Numeric |
| **P’ AMP)pera que la IA cause colapsoiónes or the area of the initial portion of a biphasic P waveiones de variables que se conocen esta)pera que la IA cause colapsoiónes or the area of the initial portion of a biphasic P waveiones de variables que se conocen esta)pera que la IA cause colapsoiónes or the area of the initial portion of a biphasic P waveiones de variables que se conocen esta** | mV | P’ wave amplitude | Numeric |
| **P’ DUR** | msec | P' wave duration | Numeric |
| **P’ AREA** | Ashman units  (40 msec x 0.1 mV) | Area of the terminal portion of a biphasic P wave | Numeric |
| **Q AMP** | mV | Q wave amplitude | Numeric |
| **Q DUR** | msec | Q wave duration | Numeric |
| **R AMP** | mV | R wave amplitude | Numeric |
| **R DUR** | msec | R wave duration | Numeric |
| **S AMP** | mV | S wave amplitude | Numeric |
| **S DUR** | msec | S wave duration | Numeric |
| **R’AMP** | mV | R' wave amplitude | Numeric |
| **R’DUR** | msec | R' wave duration | Numeric |
| **S’AMP** | mV | S' wave amplitude | Numeric |
| **S’DUR** | msec | S' wave duration | Numeric |
| **V.A.T.** | msec | Ventricular Activation Time is the interval from  the onset of the QRS complex to the latest positive  peak in the complex, or the latest substantial notch  on the latest peak (whichever is later) | Numeric |
| **QRS PPK** | mV | Peak-to-peak QRS complex amplitude | Numeric |
| **QRS DUR** | msec | QRS complex duration, measured from its onset to  the ST segment onset (J point) | Numeric |
| **QRS AREA** | Ashman units  (40 msec x 0.1 mV) | The area of the QRS complex | Numeric |
| **ST ON** | mV | Elevation or depression at the onset (J point) of the  ST segment | Numeric |
| **ST MID** | mV | Elevation or depression at the midpoint of the ST  segment | Numeric |
| **ST 80ms** | mV | Elevation or depression of the ST segment 80 ms  after the end of the QRS complex (J point) | Numeric |
| **ST END** | mV | Elevation or depression at the end of the ST  segment | Numeric |
| **ST DUR** | msec | ST segment duration | Numeric |
| **ST SLOPE** | degrees | ST segment slope. Slope is measured in degrees  for 25 mm/sec, 1mV/cm scaling, and can range  from -90 to +90 degrees. | Numeric |
| **ST SHAPE** | -, V, or ^ | The ST segment shape:  - = Straight  V = Concave upward  ^ = Concave downward | Categorical |
| **T AMP** | mV | T wave amplitude | Numeric |
| **T DUR** | msec | T wave duration | Numeric |
| **T AREA** | Ashman units  (40 msec x 0.1 mV) | T wave area for monophasic T waves or the area  of the initial portion of a biphasic T wave | Numeric |
| **T’AMP** | mV | T' wave amplitude | Numeric |
| **T’DUR** | msec | T' wave duration | Numeric |
| **T’AREA** | Ashman units  (40 msec x 0.1 mV) | Area of the terminal portion of a biphasic T wave | Numeric |
| **PR INT** | msec | Interval from the onset of the P wave to the onset  of the QRS complex | Numeric |
| **PR SEG** | msec | Interval from the end of the P wave to the onset of  the QRS complex | Numeric |
| **QT INT** | msec | Interval from the onset of the QRS complex to the  end of the T wave | Numeric |
| **Derived transverse QRS vector** | | |  |
| **Initial** | - Vector angle in degrees - Vector magnitude in mV | The vector for the initial (first 40 msec) transverse QRS signal | Numeric |
| **Maximum** | - Vector angle in degrees - Vector magnitude in mV | The maximum transverse QRS vector | Numeric |
| **Terminal** | - Vector angle in degrees - Vector magnitude in mV | The vector from the terminal (last 40 msec) or last part of the transverse QRS signal | Numeric |
| **Rotation** | 100 to -100 | - The direction of the vector rotation over the entire QRS complex - A positive rotation value indicates a clockwise vector rotation - A negative rotation value indicates a counterclockwise vector rotation - A larger magnitude indicates a higher confidence in the rotation estimate | Numeric |
| **Frontal/Horizontal plane axis parameters** | | |  |
| **P** | Degrees or indeterminate | Mean P wave axis | Numeric |
| **I:40** | Degrees or indeterminate | Initial 40ms QRS complex axis | Numeric |
| **QRS** | Degrees or indeterminate | Mean QRS complex axis | Numeric |
| **T:40** | Degrees or indeterminate | Terminal 40 msec QRS complex axis | Numeric |
| **ST** | Degrees or indeterminate | Mean ST wave axis | Numeric |
| **T** | Degrees or indeterminate | Mean T wave axis | Numeric |
| **Global measurements** | | |  |
| **Mean Ventr Rate** | Beats per minute | Representative ventricular rate for the entire ECG | Numeric |
| **Mean PR Int** | msec | Representative PR interval for the entire ECG | Numeric |
| **Mean PR Seg** | msec | Representative PR segment for the entire ECG | Numeric |
| **Mean QRS Dur** | msec | Representative QRS duration for the entire ECG | Numeric |
| **Mean QT Int** | msec | Representative QT interval for the entire ECG | Numeric |
| **Mean QTc** | msec | Representative QT interval adjusted for heart rate | Numeric |
| **QT Dispersion** | msec | Difference between the longest and the shortest QT interval in the entire ECG | Numeric |

Abbreviations: msec=milliseconds, mV=millivolts
