# Supplementary material for: Optimizing ECG to detect echocardiographic left ventricular hypertrophy with computer-based ECG data and machine learning
Source: PLoS One. 2021 Nov 30;16(11):e0260661. doi: 10.1371/journal.pone.0260661 (PMC8631676; doi:10.1371/journal.pone.0260661)
Supplement: Attachment — Submitted filename: Response to Reviewers.docx [file pone.0260661.s002.docx]

**Response to Reviewers**

**Journal Requirements**

When submitting your revision, we need you to address these additional requirements.

1. Please ensure that your manuscript meets PLOS ONE's style requirements, including those for file naming. The PLOS ONE style templates can be found at 
<https://journals.plos.org/plosone/s/file?id=wjVg/PLOSOne_formatting_sample_main_body.pdf> and 
<https://journals.plos.org/plosone/s/file?id=ba62/PLOSOne_formatting_sample_title_authors_affiliations.pdf>.

**Author Response:** We followed your instructions and corrected as necessary.

2. In ethics statement in the manuscript and in the online submission form, please provide additional information about the patient records used in your retrospective study. Specifically, please ensure that you have discussed whether all data were fully anonymized before you accessed them and/or whether the IRB or ethics committee waived the requirement for informed consent. If patients provided informed written consent to have data from their medical records used in research, please include this information.

**Author Response:** We included the next sentence: Local Ethics committee waived the requirement for informed consent as this is a retrospective study.

3. We note that you have stated that you will provide repository information for your data at acceptance. Should your manuscript be accepted for publication, we will hold it until you provide the relevant accession numbers or DOIs necessary to access your data. If you wish to make changes to your Data Availability statement, please describe these changes in your cover letter and we will update your Data Availability statement to reflect the information you provide

**Author Response:** We uploaded the dataset into a free online repository. The website is <https://figshare.com>, and the DOI is 10.6084/m9.figshare.16727524.

[Note: HTML markup is below. Please do not edit.]

**Reviewers' comments**

Reviewer #1: The authors have conducted a useful case control study to validate the CHCM algorithm that they have developed. While it has limitations in its diagnostic accuracy relative to convolutional neural networks, there are advantages to having a simple system that does not have a black box algorithm. The authors should be commended for their work in optimizing the CHCM model. I have a few revision suggestions which related to methodology and results. There are also some grammatical errors/clarifications which I have noted. I have included these as minor revisions.

Major Content Queries

1) There is now substantial evidence indicating the pitfalls of using BSA alone in indexing LVM. Did you consider using other indexation methods, such as height2.7 (De Simone et. al.) or fat free mass? Were alternative indexation methods considered to account for obesity, particularly given both of your population cohorts (LVH negative and LVH positive) were overweight? BSA has been shown to be a poor indexation method in the setting of obesity.

**Author Response:** This is a good observation. We indexed with the Mostellers formula to directly compare the results to our previous paper ([doi.org/10.1371/journal.pone.0232657](https://doi.org/10.1371/journal.pone.0232657)). In the future, we will explore if indexation by height ^2.7^ helps to predict Echo-LVH with the ECG. We added the next sentence in the limitation section (Page 24, line 406-409): "Also, we use the Mosteller formula to obtain BSA to indexed LVM, which is not optimal in the setting of the obese population. The following study will search for the best indexation method to maximize the ECG's diagnostic sensitivity for Eco-LVH".

2) Did you look into the impact of increased body size on ECG voltage criteria, particularly given your population had a mean BMI of 28.7 m^2^?.

**Author Response**: Yes. We previously described how 22 ECG criteria for left ventricular hypertrophy are affected by age, gender, BMI, Echo-LVH, and ischemic heart disease defined by Echo. The paper is published here: DOI: [10.1080/00015385.2020.1721718](https://doi.org/10.1080/00015385.2020.1721718). We could normalize the ECG criteria voltage to BMI, but we wanted to create a model that does not need anthropometric data. For sure, this will be important in the future if we're going to optimize ECG prediction capabilities fully.

3) Line 131 (methods) -> no formula is listed here for body surface area. Which calculation did you use and what was the rationale for choosing this method? It is important to note that formulae such as Du Bois are based on a very small cohort of 9 patients over 80 years ago, and as such have been discouraged in more recent literature for indexing body size variables.

**Author Response**: Thank you for this observation. We used Mosteller formula (BSA = √ (Weight x Height) / 3600) which is in page 7 line 132. We calculated using Mosteller's because we wanted to compare our previous paper (doi.org/10.1371/journal.pone.0232657) directly. Also, most large populations studies reporting LV mass have indexed to BSA and not for height. However, we will surely explore the idea of indexing LVM by height in our future studies. It would be relevant to know which indexing method is the most useful in terms of diagnostic accuracy for Echo-LVH.

4) Line 233 What was the variance (e.g. standard deviation) that you used in your sample size calculation? What outcome measure is the delta of 0.1 referring to?.

**Author Response**: The variance in our sample calculation was 10%, and we calculated the sample based on the reported sensitivity of classical criteria (around 40%). The sample size calculation was precise as our previous work ([doi.org/10.1371/journal.pone.0232657](https://doi.org/10.1371/journal.pone.0232657)). Page 12, lines 233-235, shows the sample size calculation.

5) Were you able to explore whether certain patient characteristics affected your CHCM model’s accuracy, sensitivity or specificity? For example, was CHCM better/worse based on gender, BMI or the presence of IHD?.

**Author Response**: We explored this rationale but did not find significant differences in the CHCM diagnostic accuracy for detecting these specific characteristics. We concluded that this is because the C5.0 is an algorithm that is sensitive to different types of training data. The CHCM model is trained in an overweight population, and some of them have ischemic heart disease. This lets us find the variable PPK_QRS as a significant Echo-LVH predictor because PPK_QRS is not affected by ischemia in contrast to the false negatives produced using Cornell or other state-of-the-art ECG criteria that use S wave or R wave voltage.

Minor grammatical/syntax queries

Line 21 (Abstract) -> Rather than “(ECHO-LVH) produces cardiovascular mortality”, the authors may consider rewording this e.g. “independent predictor of mortality”
Line 85 (background) -> change “produces” to “produce”
Line 88 -> change “are exploring” to “have explored”
Line 204 -> change “de” to “the”
Line 211 -> change “inhomogeneity” to “in homogeneity”
Line 213-> change “cut-values” to “cut-off values”
Lines 216/217 -> this sentence is difficult to understand, suggest breaking into smaller sentences “Also, decision trees can continue to grow indefinitely as more and more predictors are presented to the C5.0 algorithm -this is called overfitting- and created precise predictions in new datasets”:

**Author Response**: All minor queries are corrected. To correct the latter, we added the following sentence: "Also, decision trees can continue to grow indefinitely -this is called overfitting- and compromises generalization to new information.". This can be found on pages 11, lines 216 and 217.


Reviewer #2: This paper presents a method that detects echocardiographic left ventricular hypertrophy (Echo-LVH) using electrocardiogram (ECG). The authors used the Philips DXL-16 algorithm to extract 458 ECG parameters. They then used the C5.0 machine learning (ML) algorithm to automatically find the most relevant parameters associated with Echo-LVH and used them to train a predictive model. The proposed model was evaluated in a retrospective manner on 439 patients and compared with other conventional detection approaches. The results show that the proposed predictive model had similar accuracy to other approaches using a new and fewer sets of parameters.

The proposed cardiac hypertrophy computer-based model (CHCM) is an extension of the authors’ prior work, Cardiac Hypertrophy Model which uses manually selected ECG parameters including ST ECH-LVH, voltage-left atrial enlargement LVH and voltage-duration LVH coupled with C5.50 ML algorithm. To me, it was difficult to clearly understand the contribution of CHCM in comparison to existing models. Apart from being able to leverage the digital data extracted from Phillips DXL-16 algorithm, there seems to be limited new insights and findings introduced from this paper. The in-depth discussions about derived ECG parameters and their corresponding roles in detecting Echo-LVH in comparison to existing algorithms would be important.

**Author Response:** Although the CHCM does not solve the problem of the low accuracy of the ECG for Echo-LVH, it has some advantages over classical ECG criteria (i.e. Cornell, Sokolov). For example, the CHCM uses both depolarization and repolarization to classify patients. This is relevant as patients with LVH diagnosed by voltage (depolarization) or by repolarization abnormalities have different mortality rates. This was demonstrated in the Atherosclerosis Risk in Communities (ARIC) study were they found that the individual components of the Romhilt-Estes LVH score differ in their prediction of cardiovascular events. Classical criteria such as Cornell and Sokolov totally ignore repolarization abnormalities. Also, in contrast to Romhilt-Estes and our previous criteria, the CHCM does not need auricular information (P wave), this means that the CHCM uses only ventricular ECG data and not also auricular data, this seems to me to be a more logical approach. Also, most criteria does not use T wave amplitude, even though T wave is relevant in LVH. We added the following sentence in pages 22 and 23 lines 373-378: “The CHCM has some advantages over classical ECG criteria (i.e., Cornell, Sokolov). For example, the CHCM uses both depolarization and repolarization abnormalities. The Atherosclerosis Risk in Communities (ARIC) study demonstrated that the individual components (both depolarization and repolarization abnormalities) of the Romhilt-Estes LVH score differ in predicting cardiovascular events. Therefore, it is crucial to detect these two phenotypes (voltage or depolarization and repolarization Echo-LVH) ”. We added the reference number 37.

Other detailed comments:

1. Page 6, line 101: Inform --> Informed
2. Page 6, line 108: STARD --> not defined. **Author Response**: We added in page 6 line 108-109 the next sentence: “We followed the STARD methodology for reporting studies of diagnostic accuracy and international guidelines for the development of Machine Learning (ML) models”
3. Page 6, line 109: ML --> Machine Learning (ML)
4. Page 9, line 172: most of the sample --> what is sample in this context? **Author Response:** We corrected this in page 13, lines 245-248.
5. Page 13, line 244-249: The sentences in this paragraph are redundant. **Author Response:** Thanks for the comment. With the total sample this study reached a power of 99%. The first sample was split into two groups: 70%(n=307) and another for testing 30%(n=132) of the CHCM. A second sample was included for external validation [156, 35.5%]). This sentence is in page 13, line 245-248.
6. Page 15, Table 2: Total sample --> Total Samples
7. Page 15, Table 2: why is the number of total samples 321? Why not 439, consistent with Table 1?. **Author Response**: Table 2 shows only 321 patients because there are 26.9% of missing values in comorbidities. Missing values in comorbidities were allowed but patients with missing values in Echo, and ECG data were excluded. We wrote a footnote in table 2 that describes why the sample in this table is 321 and not 439 (“Missing completely of random values of comorbidities were 26.9% of the total sample. We performed complete case analyses).
8. Page 17, line 309: more accuracy --> higher accuracy
9. Page 23, line 398: ‘However, the CHCM is half the size of the Cardiac Hypertrophy model, which gives a more biological coherent model based on the principle of parsimony-the simpler explanation is the most probable’ --> this seems to be an over justification that explains nothing much. Please be specific and articulate the differences. **Author Response**: We added the next sentence: “However, the CHCM is half the size of *the Cardiac hypertrophy model,* making it a lot simpler and easier to use”

**Author Response:** all minor queries have been corrected.
