# Supplementary material for: Optimizing ECG to detect echocardiographic left ventricular hypertrophy with computer-based ECG data and machine learning
Source: PLoS One. 2021 Nov 30;16(11):e0260661. doi: 10.1371/journal.pone.0260661 (PMC8631676; doi:10.1371/journal.pone.0260661)
Supplement: Attachment — Submitted filename: Response to Reviewers.docx [file pone.0260661.s003.docx]

**Response to reviewers**

**Editor Comments:** Thank you for amending your manuscript. I believe that the manuscript became much stronger. However, as one of the reviewers pointed out, we would recommend seeking assistance from a native English speaker with scientific writing skills in order to help our readers.

**Response:** Thank you for all your suggestions. Thank you so much for being so helpful in creating a better version of our manuscript.

**Reviewers' comments:**

**Reviewer #1:** The authors have made appropriate major changes, however none of the extensive grammatical issues identified have been addressed. As a result, the paper is not written in standard english. I would suggest that the authors seek assistance from an English grammer expert to proof read their manuscript prior to resubmission.

**Response:** We sought assistance from an English grammar expert, and now the proof is written in standard English. Thank you.

**Reviewer #2:** The authors have addressed all the comments satisfactorily. The contributions of the paper have been articulated.

**Response:** Thanks.
